# Ameliorative Effect of *Bacillus subtilis* on Growth Performance and Intestinal Architecture in Broiler Infected with Salmonella

**DOI:** 10.3390/ani9040190

**Published:** 2019-04-23

**Authors:** Alaeldein M. Abudabos, Muttahar H. Ali, Mohammed A. Nassan, Ahmad A. Saleh

**Affiliations:** 1 Department of Animal Production, College of Food and Agriculture Sciences, King Saud University, P.O. Box 2460, Riyadh 11451, Saudi Arabia; muttahar2013@gmail.com; 2 Department of Pathology, Faculty of Veterinary Medicine, Zagazig University, Zagazig 44511, Egypt; moh_nassan@yahoo.com; 3 Department of Poultry Production, Faculty of Agriculture, Kafrelsheikh University, Kafr El-Sheikh 333516, Egypt; a_saleh2006@yahoo.com

**Keywords:** broiler, growth, intestinal villi, *Salmonella*

## Abstract

**Simple Summary:**

Salmonellosis is a dangerous disease in broilers that causes huge economic losses. We assumed that instead of antibiotics, a Bacillus-based probiotic may serve as an alternative to alleviate the negative effects of *Salmonella* infection. A control group with no feed additive, a positive control supplemented with a standard antibiotic and two groups that were supplemented with different strains and levels of *Bacillus subtilis* were the experimental animals of the present study. It was revealed that supplementation of probiotic bacteria induced similar results in terms of feed intake, body weight gain and feed efficiency in comparison with the group treated with antibiotics. In addition, the dimensions of intestinal villi were also improved in the probiotic-treated birds. As concluded from the results of the present study, probiotic bacteria could be used as an alternative to antibiotics against *Salmonella* in broilers.

**Abstract:**

A total of 600 day-old broiler chicks (Ross 308) confirmed for the absence of *Salmonella* were randomly allocated to five treatments each with 10 replicates: negative control (basal diet only); positive control (basal diet) + infected with *Salmonella*; T1, *Salmonella* infected + avilamycin; T2, *Salmonella* infected + *Bacillus subtilis* (ATCC PTA-6737; 2 × 10^7^ CFU/g) and T3, *Salmonella* infected + *B. subtilis* (DSM 172999; 1.2 × 10^6^ CFU/g). The results revealed that feed intake (FI) and body weight (BW) were significantly (*p* < 0.01) lower in T1 compared to T2. The feed conversion ratio (FCR) was significantly (*p* < 0.01) lower in T2 and T3 compared to other treatments. Similarly, the performance efficiency factor (PEF) was also significantly (*p* < 0.01) higher in T2 and T3 compared to positive control. Villus height was significantly (*p* < 0.01) higher in T2 compared to all other treatments. However, villus width and surface area were significantly (*p* < 0.01) higher in T1. In conclusion, dietary supplementation with *B. subtilis* improved growth and intestinal health by reversing the negative effects of Salmonellosis.

## 1. Introduction 

In the modern broiler industry, antimicrobials used as growth promoters are among the most popular synthetic agents used in poultry production for the improvement of feed efficiency and the reduction of microbial pathogenesis [1,2]. Antimicrobials as an additive have produced promising results; however, their regular use has caused drug resistance and residues in eggs and meat [3]. Under such circumstances, many countries are considering a strict ban or have already banned (European Union) the use of Antimicrobial growth promoters (AGPs) [4,5,6]. Therefore, there is a necessity to find suitable alternatives that could replace AGPs. Recently natural products have gained special interest, since they improve growth performance and reduce mortality rates as an effect of infectious agents [7,8,9].

*Salmonella* is one of the most important poultry diseases causing heavy economic losses through stunted growth and increased mortality rates [6,8]. Incidences of *Salmonella* are most frequent during the starter phase, since the immune system of the chick is not well developed [9]. Chickens are frequently exposed to *Salmonella* during their life and micro-organisms can be readily transferred to humans through the consumption of poultry meat, causing severe gastrointestinal symptoms [10]. A number of practices are used to reverse the symptoms of salmonellosis in broilers including the use of probiotics [9,11]. Probiotics are used in poultry production due to the wide range of their positive effects [11]. Probiotics are now considered an alternative to antibiotics and added in the animals’ diet as a microbial supplement [12]. It has been reported that probiotics enhance growth, provide protection against a wide range of pathogens and improve immunity [11,12,13]. *Bacillus subtilis *is naturally isolated from the gut of chickens and it is known to produce antimicrobial substances such as surfactants [11]. Recently, it was reported that *B. subtilis* improved the growth and antioxidant status in broilers exposed to *Salmonella* [8]. In the literature, positive effects of the two strains of *B. subtilis* have been reported; however, their comparative effects have not been described. The aim of the present study was to evaluate the effects of two different strains of *B. subtilis* on the performance and intestinal health of *Salmonella*-infected broilers during the starter phase. 

## 2. Methods

### 2.1. Animals and Feeding Practices and Randomization

A total of 600 day-old broiler chicks (Ross 308) were randomly divided into five treatments (10 replicates and 12 birds per replicate). On arrival, all chicks were confirmed for the absence of *Salmonella*. The experiment was carried out in an environmentally controlled closed poultry unit. Straw was used as bedding material on the concrete floor. Initially the temperature was set to 31 °C and gradually decreased to a thermoneutral temperature of 22–24 °C and a relative humidity of 70%. An automated exhaust fan drew outside air in at 45.8 m^3^/min. The photoperiod was maintained at 23:1 L:D at the intensity rate of 20 lux using cool light fluorescent tubes. The stocking density was maintained at 50 kg/m^2^. 

Broiler chicks were raised according to the recommendations of the Ross guide. A standard starter (0–15) diet with isocaloric and isonitrogenous contents was offered in a mash form based on corn-SBM and was formulated to meet the requirements of the broilers (Table 1). On day 1, chicks received one of five treatments randomly as follows: negative control (basal diet); positive control (basal diet) + infected with *Salmonella enterica*, subspecies *typhimurium*; T1, infected with *Salmonella* + avilamycin (0.2 g/kg); T2, infected + probiotics that have viable spores of *B. subtilis* (ATCC PTA-6737; (2 × 10^7^ CFU/g) and T3, infected + *B. subtilis* (DSM 17299; 1.2 × 10^6^ CFU/g). 

### 2.2. Challenge Inoculum 

On the second day, all groups apart from the negative control were orally administered with a 3 × 10^9^ live culture of *Salmonella enterica* subspecies *typhimurium* as described by Abudabos et al. [9]. 

### 2.3. Growth Performance

Growth performance parameters such as body weight (BW), feed intake (FI) and feed conversion rate (FCR) were recorded at five-day intervals. The BW was measured on an electronic digital balance with a sensitivity of 0.1 g (Berkley, Columbia, SC, USA). The production efficiency factor (PEF) was calculated as described by Abudabos et al. [6]. 

### 2.4. Sitophological Measurements

Histological study of intestinal tissue was conducted as described by Rahman et al. [14]. On day 15, 10 birds per treatment were randomly selected. For histological studies, about 2 × 2 cm^2^ long samples from the proximal portion of the ileum were collected, fixed and then embedded in paraffin. The tissues were sectioned to roughly 5 mm small pieces and stained (hematoxylin and eosin). Ten well-oriented villi per sample were selected and measured using a simple microscope (Olympus) connected to an image analysis system. 

### 2.5. Statistical Analysis 

All statistical analyses were performed using the Statistical Analysis System [15]. Means were compared by the method described by Steel and Torrie [16] and a significant level was obtained by the Duncan multiple-range test [17]. 

### 2.6. Ethical Approval 

The study was approved by the Committee on Care and Use of Animals, King Saud University, Saudi Arabia (1127/18/DAP). 

## 3. Results

### 3.1. Growth Performance 

The results of growth performance for the control and experimental groups are provided in Table 2. Feed intake was significantly (*p* < 0.01) higher in T2 compared to T1. Similarly, BW was significantly (*p* < 0.01) higher in T2 compared to T1 and the positive control. As a result, FCR was significantly (*p* < 0.01) lower in T2 and T3 compared to the positive control. Similarly, PEF was also significantly (*p* < 0.01) higher in T2 and T3 compared to the positive control. Although PEF in T1 was not significantly different from T2 and T3, values were slightly better in T2 and T3 compared to T1. 

### 3.2. Intestinal Histology

The effects of *B. subtilis* on histological structures of broiler chickens are presented in Table 3. Villus height was significantly (*p* < 0.01) higher in T2 compared to all other treatments. However, villus width and surface area were significantly (*p* < 0.01) higher in T1 compared to the positive control group. Villus width was not statistically significant between the negative control and T1 groups. 

## 4. Discussion

In the current study, growth performance and intestinal histological parameters were improved in the probiotic-treated birds infected with *Salmonella*. The results were similar to those of the antibiotic-treated birds. Probiotics are considered one of the viable alternatives to antibiotics, particularly in view of the recent ban of regular use of AGPs in poultry diet [6]. In the present study, the growth performance and intestinal architecture were significantly deteriorated in the *Salmonella*-infected birds. Reduced growth and lesion in the intestinal villi are some of the most prominent signs of salmonellosis, leading to heavy economic losses [8]. 

Interestingly, the results of the probiotic-treated birds were comparable to those of the birds fed antibiotic supplements. The improved growth performance of broilers infected with different kinds of pathogens such as *Clostridium* and *Salmonella* in response to supplementation of *B. subtilis* or phytogentics has been published recently [8,9]. The positive effects of probiotics are well documented, e.g., improved performance (body weight gain and feed conversion rate); enhanced immune response and healthy intestine [11]. The effects of the two types of probiotics on the performance of the birds were not significantly different. The efficacy of probiotic use is linked to genetics, nutritional status, frequency, dose, specificity of the strain, survival and stability [11]. A number of mechanisms through which probiotics produce positive effects are involved, such as the reduction of intestinal pH, production of volatile fatty acids and suppression of pathogenetic bacteria through competitive exclusion [18]. 

As indicated in the current study, villus dimensions were restored as an effect of probiotic supplementation. Dietary probiotics have been shown to enhance the intestinal microbiome in a positive direction [8,9]. The intestinal villi secrete different kinds of defensive mucins such as MUC2 and MUC3 from the goblet cells [11]. In addition, probiotic bacteria improve the humoral and cellular immunity through increased production of delayed hypersensitivity, respiratory burst of macrophages, antibody production, natural killer cells, interleukins, cytokines, antibody-secreting cells and T lymphocytes [11,18]. 

## 5. Conclusions 

Dietary supplementation with *B. subtilis* improved the growth performance and gut health of *Salmonella*-infected broiler chickens. 

## Figures and Tables

**Table 1:** Dietary composition of broiler chicken starter diets.

Ingredient (%)	Starter Phase
Yellow corn	57.39
Soybean meal	27.00
Palm oil	2.20
Corn gluten meal	8.80
Dicalcium phosphate	2.30
Ground limestone	0.70
Choline chloride	0.05
DL-methionine	0.10
L-lysine	0.39
Salt	0.40
Threonine	0.17
V-M vitamins-minerals premix ^1^	0.50
**Analyses**	
ME Metabolizable energy, kcal/kg	3000
Crude protein, %	23.0
Non phytate P, %	0.48
Calcium, %	0.96
D. Lysine, %	1.28
D. Methionine, %	0.60
Sulfur amino acids, %	0.95
Threonine, %	0.86

^1^ Vitamin-mineral premix contains the following per kg: vitamin A, 2,400,000 IU; vitamin D, 1,000,000 IU; vitamin E, 16,000 IU; vitamin K, 800 mg; vitamin B1, 600 mg; vitamin B_2_, 1600 mg; vitamin B_6_, 1000 mg; vitamin B_12_, 6 mg; niacin, 8000 mg; folic acid, 400 mg; pantothenic acid, 3000 mg; biotin 40 mg; antioxidant, 3000 mg; cobalt, 80 mg; copper, 2000 mg; iodine, 400; iron, 1200 mg; manganese, 18,000 mg; selenium, 60 mg; zinc, 14,000 mg.

**Table 2:** Means ± SE of feed intake (FI), body weight (BW), feed conversion ratio (FCR), body weight (BW) and performance efficiency factor (PEF) for the cumulative starter period (0 to 15 days of age).

Treatment	FI (g)	BW (g)	FCR (g:g)	PFE
Negative control	437.0 ^a,b^	346.9 ^a^	1.259 ^d^	196.6 ^a^
Positive control	426.8 ^a^	282.9 ^c^	1.511 ^a^	134.6 ^d^
T1	391.4 ^b^	281.9 ^c^	1.390 ^b^	150.5 ^b,c,d^
T2	440.8 ^a^	321.5 ^a,b^	1.374 ^b,c^	171.3 ^b^
T3	416.3 ^a,b^	314.0 ^b^	1.329^c^	165.8 ^b,c^
SEM±	11.46	9.12	0.019	8.19
*p* value	0.0005	0.0001	0.0001	0.0001

^a,b,c^ Means within a column differ significantly (*p* < 0.01). T1, infected + avilamycin at a rate of 0.2 g/kg; T2, infected + probiotics that have viable spores (2 × 10^7^ CFU/g) of *Bacillus subtilis* (ATCC PTA-6737); T3: infected + *B. subtilis* (DSM 17299;1.2 × 10^6^ CFU/g).

**Table 3:** Means ± SE of villi height (L), width (W) and villi total area (TA) of ileum in broiler chickens at 15 days.

Treatment	Villus Height (µm)	Villus Width (µm)	Total Area (mm^2^)
Negative control	439 ^c^	76.7 ^a^	0.100 ^b,c^
Positive control	425 ^c^	64.17 ^b^	0.085 ^d^
T1	544 ^b^	73.9 ^a^	0.124 ^a^
T2	614 ^a^	57.6 ^b,c^	0.110 ^b^
T3	562 ^b^	61.1 ^b,c^	0.108 ^b,c^
SEM±	11.96	2.79	0.004
*p* value	0.0001	0.0001	0.0001

^a,b,c^ Means within a column differ significantly (*p* < 0.01). T1, infected + avilamycin at the rate of 0.2 g/kg; T2, infected + probiotics that have viable spores (2 × 107 CFU/g) of *B. subtilis* (ATCC PTA-6737); T3: T3, infected + *B. subtilis* (DSM 17299 1.2 × 10^6^ CFU/g).
